# The need for novel strategies to address postoperative pain associated with cardiac surgery: A commentary and introduction to “SMArTVIEW”

**DOI:** 10.1080/24740527.2019.1603076

**Published:** 2019-07-30

**Authors:** Carley Ouellette, Shaunattonie Henry, Andy Turner, Wendy Clyne, Gill Furze, Marissa Bird, Karla Sanchez, Judy Watt-Watson, Sandra Carroll, PJ Devereaux, Michael McGillion

**Affiliations:** a Faculty of Health Sciences, School of Nursing, McMaster University, Hamilton, Ontario, Canada; b Perioperative & Digital Health Department, Population Health Research Institute, Hamilton, Ontario, Canada; c Faculty of Health and Life Sciences, Coventry University, Coventry, UK; d Hope for the Community CIC, Coventry, UK; e Lawrence S. Bloomberg Faculty of Nursing, University of Toronto, Toronto, Ontario, Canada

**Keywords:** cardiac surgery, vascular surgery, remote automated monitoring, nursing, self-management

## Abstract

**Background**: With coronary heart disease affecting over 2.4 million Canadians, annual cardiac and major vascular surgery rates are on the rise. Unrelieved postoperative pain is among the top five causes of hospital readmission following surgery; little is done to address this postoperative complication. Barriers to effective pain assessment and management following cardiac and major vascular surgery have been conceptualized on patient, health care provider, and system levels.

**Purpose**: In this commentary, we review common patient, health care provider, and system-level barriers to effective postoperative pain assessment and management following cardiac and major vascular surgery. We then outline the SMArTVIEW intervention, with particular attention to components designed to optimize postoperative pain assessment and management.

**Methods**: In conceptualizing the SMArTVIEW intervention design, we sought to address a number of these barriers by meeting the following design objectives: (1) orchestrating a structured process for regular postoperative pain assessment and management; (2) ensuring adequate clinician preparation for postoperative pain assessment and management in the context of virtual care; and (3) enfranchising patients to become active self-managers and to work with their health care providers to manage their pain postoperatively.

**Conclusions**: Innovative approaches to address these barriers are a current challenge to health care providers and researchers alike. SMArTVIEW is spearheading this paradigm shift within clinical research to address barriers that impair effective postoperative pain management by actively engaging health care providers and patients in an accessible format (i.e., digital health solution) to give primacy to the need of postoperative pain assessment and management following cardiac and major vascular surgery.

## RÉSUMÉ

**Contexte:** Alors que la maladie cardiaque coronarienne touche plus de 2,4 millions de Canadiens, les taux annuels de chirurgie cardiaque et de chirurgie vasculaire majeure sont en augmentation. Bien que la douleur postopératoire non soulagée compte parmi les cinq principales causes de réadmission à l’hôpital après une chirurgie, peu de choses sont faites pour remédier à cette complication postopératoire. Les barrières à l’évaluation et à la prise en charge efficaces de la douleur après une chirurgie cardiaque ou une chirurgie vasculaire majeure ont été conceptualisées aux niveaux du patient, du prestataire de soins et du système.

**But**: Dans ce commentaire, nous examinons les barrières à l’évaluation et à la prise en charge efficace de la douleur postopératoire après une chirurgie cardiaque et une chirurgie vasculaire majeure au niveau du patient, du prestataire de soins et du système. Nous présentons ensuite l’intervention SMArTVIEW, en portant une attention particulière aux composantes conçues pour optimiser l’évaluation et la prise en charge de la douleur postopératoire.

**Méthodes**: Au moment de conceptualiser l’intervention SMArTIEW, nous avons cherché à remédier à un certain nombre de ces barrières à partir des objectifs de conception suivants : 1) orchestrer un processus structuré pour l’évaluation et la prise en charge de la douleur postopératoire régulière; 2) assurer une préparation adéquate des cliniciens pour l‘évaluation et la prise en charge de la douleur postopératoire dans un contexte de soins virtuels; et 3) affranchir les patients afin qu’ils puissent activement prendre en charge leur douleur postopératoire et travailler de concert avec les prestataires de soins.

**Conclusions**: Les approches innovantes pour remédier à ces barrières sont un défi constant, tant pour les prestataires de soins que pour les chercheurs. SMArTVIEW est à l’avant-garde de ce changement de paradigme dans la recherche clinique qui a pour but de remédier aux barrières qui nuisent à la prise en charge efficace de la douleur postopératoire en engageant activement les prestataires de soins et les patients dans un format accessible (i.e. solution de santé numérique), afin que la primauté soit accordée à la nécessité d’évaluer et de prendre en charge la douleur postopératoire après une chirurgie cardiaque ou une chirurgie vasculaire majeure.

## Introduction

With coronary heart disease affecting over 2.4 million Canadians, annual cardiac and major vascular surgery rates are on the rise. In 2014, the median annual number of cardiac surgery cases across Canada was estimated at 30 000.^1^ The 2017 Canadian Institutes of Health Information’s *Cardiac Care Quality Indicators Report* concluded that though Canada is performing well on quality indicators for cardiac and major vascular surgery, several opportunities for quality improvement remain, including unrelieved acute postoperative pain.^2^ The national average 30-day readmission rate is 9.5%; provincial readmission rates vary between 6.5% and 11.9%.^2^ Across cardiac surgeries, average readmission rate after isolated coronary artery bypass graft is highest at 6.9%.^2^ Irrespective of surgical type, unrelieved postoperative pain is among the top leading drivers of readmissions.^3^ A prospective, multicenter cohort study (10 centers, 5185 patients) by the Joint National Institutes of Health–Canadian Institutes of Health Research Cardiothoracic Surgical Trials Network, for example, found that nearly one in five patients was readmitted within 60 days after discharge; the leading drivers of readmission between 30 and 60 days were volume overload, surgical site infection, and pain.^3^

As outlined by the International Association for the Study of Pain (IASP), pain is defined as a subjective, multidimensional experience with sensory, cognitive, and affective components.^4^ Mounting evidence has demonstrated that unrelieved postoperative pain is associated with poor sleep hygiene and fatigue,^5,6^ anxiety and depressive disorders,^7–9^ poor perceived self-efficacy, as well as poor self-rated health.^5,10^ In addition, unrelieved postoperative pain is associated with a number of complications. For example, prospective studies have found that unrelieved acute pain in the severe range (i.e., Numeric Rating Scale [NRS] ≥ 7/10) at postoperative day 3 is associated with increased risk of transition to chronic postsurgical pain (adjusted odds ratio = 2.67, 95% confidence interval, 1.74–4.11).^11^ Though effective postoperative pain assessment and management are key to optimal recovery, little is done to address unrelieved acute pain as a leading driver of readmission.^3^ Barriers to effective pain assessment and management following cardiac and major vascular surgery have been conceptualized on patient, health care provider, and system levels.^12–15^

In 2015, our team released a position statement in the *British Journal of Cardiology*, arguing the need for a digital health connected care strategy—from hospital to home—that would address leading drivers of readmission following cardiac and major vascular surgery.^16^ In 2018, we launched an international, randomized controlled trial (HiREB#3641) of a multicomponent digital health intervention entitled “TecHnology Enabled Self-MAnagemenT—VIsion for remote automated patient monitoring and EmpoWerment following Cardiac and VasculaR surgery (THE SMArTVIEW CoVRed), more commonly referred to as SMArTVIEW.^17^ Currently underway, the trial (*N* = 800) was designed to examine the impact of the SMArTVIEW intervention on a primary composite of 45-day hospital readmission, emergency department visits, and urgent care center visits in Canada and the United Kingdom. The impact of the intervention on an array of postoperative adverse events is also being examined, including unrelieved moderate to severe acute postoperative pain (i.e., NRS ≥ 4/10).

In this commentary, we review common patient, health care provider, and system-level barriers to effective postoperative pain assessment and management following cardiac and major vascular surgery. We then outline the SMArTVIEW intervention, with particular attention to components designed to optimize postoperative pain assessment and management.

## Patient-level barriers

### Inadequate preparation to self-manage

Patients are ill-prepared to self-manage pain both in hospital and at home. While in hospital, patients are often unclear of their own role in pain management and lack knowledge, skills, and confidence about how to communicate their pain experience and symptoms to clinicians.^12^ For example, in a survey of elderly surgical patients (*N* = 125), Brockopp et al.^18^ found that 53% of patients disagreed with the following statement: “When people are in pain due to surgery, it is better to take pain medication regularly rather than just when they hurt” and 31% agreed that “in the hospital, doctors are the only people who can help patients with their pain.” Whether patients lack knowledge, have preconceived ideas or attitudes, or assume to be passive recipients of pain relief, challenges persist in engaging patients as active self-managers immediately following surgery.^19,20^

This problem persists when patients are discharged home. Following discharge, patients are typically instructed to follow up with their primary care provider 1 week following surgery and with their surgeon within 6 to 8 weeks.^21^ However, patients often struggle to manage their recovery during this care transition period and lack the basic knowledge and skills to self-manage their pain at home, including both pharmacological and nonpharmacologic strategies.^13^ Data from the Canadian CARDpain (Chronic Postoperative Pain After Coronary Artery Bypass Graft Surgery) study (*N* = 1247) demonstrated that over 50% of patients, across three provinces, reported significant bodily pain and pain-related interference with family and home responsibilities, recreation, and returning to employment during recovery up to 1 year following surgery.^11^

### Problematic pain-related beliefs

Cumulative evidence supports that outdated beliefs and inaccurate information contribute to suboptimal pain assessment and management practices.^12,14,22,23^ For example, patients often expect to endure pain following surgery and will routinely underreport their pain in order to be “good patients” and avoid analgesics, even when the intensity of their postoperative pain is in the moderate to severe range (NRS ≥ 4/10).^22,23^ Cogan et al.^24^ used the Barriers Questionnaire to examine pain-related beliefs in a cohort of 564 patients scheduled for cardiac surgery. Among those who responded (n = 379), 60% reported the personal belief that “good patients avoid talking about pain”; 54% agreed that “pain medication should be saved in case pain worsens”; and 33% agreed that patients can easily become addicted to pain medication following surgery. As Cogan et al.^24^ concluded, minimal advances have been made in reframing pain-related misconceptions and misbeliefs, which remain prevalent in postoperative settings to date.

Evidence from randomized controlled trials has found that perioperative educational interventions, designed to target pain-related misbeliefs among patients undergoing cardiac surgery, consistently yield small to no effects in terms of improving patient pain-related knowledge, skills, and pain management outcomes.^12,25–29^ In 2004, Watt-Watson et al.^25^ examined the impact of a preoperative education booklet titled, “Pain Relief After Your Surgery” in order to reduce acute postoperative pain intensity and pain-related interference with recovery (*n* = 406). Compared to usual care, the intervention group did not experience improved pain management; some significant gains in terms of pain-related interference (*P* < 0.01) as well as concerns related to analgesics (*P* < 0.05) were found. A trial in 2017 by Cogan et al.^12^ tested the impact of an updated version of Watt-Watson’s et al.’s^25^ 2004 booklet, with input from pain experts across Canada (via an online portal). The updated version, including the latest content on the importance of good pain relief, asking for help, management strategies, and pain-related misbeliefs, yielded no significant impact on patients’ pain knowledge and attitudes toward pain management.

The available evidence to date supports that addressing patient-level barriers to effective pain assessment and management is complex and indeed challenging. As Bjørnnes et al.,^30^ Leegaard et al.,^20^ and others^31–35^ have argued, a confluence of factors are at play in the context of surgical care, which serves to confound efforts to optimize conditions for perioperative pain education. Some of these contextual factors have been identified at both the health professional and health system levels.

## Health care professional barriers

### Inadequate preparation

Health care providers often lack adequate preparation in terms of evidence-based best practices for postoperative pain assessment and management. For example, in a descriptive study employing a correlational, mixed between/within-subjects design, Watt-Watson et al.^36^ found that across three cardiac surgery units in Canadian university–affiliated hospitals, nurses’ (*n* = 94) pain knowledge scores were not significantly associated with nurses’ postoperative pain intensity ratings. Moreover, deficits in pain-related knowledge as well as misbeliefs about pain management were found. Patients cared for by these same nurses (*n* = 225) reported unrelieved moderate to severe postoperative pain (NRS ≥ 4/10) yet they only received 47% of their prescribed analgesic. Nearly one third of all nurses disagreed with their patients’ pain ratings more than 25% of the time, and 40% believed that their patients overstated their pain more than 25% of the time.

Since Watt-Watson et al.’s^36^ study, a number of additional studies have corroborated divergent views between nurses’ assessments and patients’ self-reports of pain experience. In a 2005 survey study administered to surgical patients (*N* = 95) and nurses (*N* = 95), statistically significant differences (*P* < 0.05) were found on all metrics, wherein nurses consistently underrated their patients’ pain scores related to pain upon movement, pain upon rest, overall pain intensity, as well as patient suffering due to pain compared to patient self-reports.^37^ Similar findings were elucidated in a 2017 cross-sectional study of surgical nurses across 73 institutions that found that only 63% of patients had self-reported pain intensity scores that were consistent with their nurses’ impressions of their pain experiences.^22^ Some potential contributing factors influencing discrepancies between nurse assessments and patient reports include how pain is conceptualized, understanding of rating scales and what is “moderate” pain on the NRS, and what methods are used by nurses to conduct pain assessments.^19,37^

Inadequate preparation of interprofessional health care providers to competently assess and manage postoperative pain has been widely cited as a problem originating at the prelicensure education level.^38–46^ For example, a survey completed in 2009 of prelicensure pain curricula across Canadian university health sciences faculties found that just one third of respondents were able to identify specific time allocated to pain content.^38^ The remainder of respondents reported having some “integrated” content that was not quantifiable in terms of hours dedicated to pain-specific teaching. A recent systematic review of 14 international studies reported similar results, with 20% of medical schools in the United States identifying less than 5 h of teaching, in total, dedicated to pain.^46^

The last two decades have seen concentrated efforts by the IASP and other groups to move prelicensure pain education forward through intensive focus on interprofessional pain curricula and related practice competencies, as well as through a shift in emphasis from didactic modes of education to experiential and contextualized learning models that are competency based.^47^ The University of Toronto interfaculty pain curriculum, for example, has been successful in delivering a mandatory 20-h interprofessional curriculum for students across six health sciences faculties since 2002, demonstrating consistent improvements in prelicensure students’ pain knowledge and beliefs scores, as well positive student evaluations related to content and process-related outcomes.^48^

Though greater attention has been focused on pain education, in-person education delivery is resource intensive.^49^ To overcome implementation barriers of face-to-face formats and resource constraints, some headway is also being made in terms of online prelicensure pain curriculum delivery.^49^ The recent Pain Education Interprofessional Resource study examined the impact of a multimedia e-learning model, based on IASP core competencies, on user experience, pain knowledge and beliefs, as well as empathic pain assessment skills.^50,51^ Pre–post evaluation (*N* = 96) found that the Pain Education Interprofessional Resource was ranked highly as a usable education platform, yielding an overall 20% improvement in student pain knowledge and belief scores.

## Health system barriers

System barriers also pose significant challenges to optimal postoperative pain assessment and management. Within hospitals, acute pain services (APS) are often considered a valuable resource to call upon in order to access pain-related expertise. Yet, such services are often underutilized in surgical populations.^52^ A 2017 survey of Canadian hospitals reported that 31 centers offer cardiothoracic surgical services, of which 70% had a designated APS. Of these centers, most stated that their APS was “rarely involved in cardiac surgical cases (p. 1236),” quantifying that APS only followed approximately 10% cardiac surgical patients.^52^

As discussed previously, unrelieved acute postoperative pain has been documented as a significant risk factor for transition to chronic post surgical pain (CPSP) following cardiac surgery. Prevalence rates for patients who underwent Coronary Artery Bypass Graft (CABG), valve replacement or combination of both who developed CPSP have been estimated at 41% (*n* = 87/212)^53^ at 12 weeks following surgery. Estimates of CPSP at 2 years following surgery are nearly 10% (*n* = 93/976)^11^ across multiple centers, thus indicating that CPSP is a serious postoperative complication that not only negatively affects patients and derails their postoperative recovery but also necessitates further access to health care services. Amoung those who develop CPSP, many will be required to endure prolonged wait times to access publicly funded expert pain-related care, if able to access specialized care at all given that many regions of Canada lack any access to multidisciplinary pain care.^54,55^

Fortunately, in recent years, greater attention has been drawn to address inadequacies and inequities in the delivery and optimization of postoperative pain management. For example, the University Health Network has recently deployed a Transitional Pain Service (TPS) that allocates resource stewardship and interdisciplinary care toward patients at risk of developing chronic pain.^56^ The TPS initiates care preoperatively and follows patients up to 6 months postoperatively. Recently, this group examined opioid weaning in a cohort of 251 patients seen by the TPS.^57^ Of these patients, 18% (*n* = 45) were patients underoing major cardiac or vascular suergery. Preliminary evaluation has shown positive result of the TPS. Average pain intensity scores were decreased by a mean of 17% and functional impairment from pain (Brief Pain Inventory–Pain Interference subscale) improved by 21% from hospital discharge to final TPS follow-up appointment. Results to date are promising, indicating that the TPS, as a specialized pain service designed to follow patients during the first 6 months after surgery, is of benefit to surgical patients in terms of opioid consumption, pain, and function-related outcomes. Health care utilization and cost effectiveness and utility analyses are needed as well to further substantiate the widespread deployment of such services.

In summary, patient, health care provider, and system-level barriers to effective postoperative pain assessment and management have been longstanding, complex problems.

In conceptualizing our design of the SMArTVIEW intervention, we sought to strategically address a number of these barriers by meeting the following design objectives^16^: (1) orchestrating a structured process for regular postoperative pain assessment and management in an accessible digital format, from hospital to home; (2) ensuring adequate clinician preparation for postoperative pain assessment and management and related problem solving in the context of virtual care; and (3) enfranchising patients to become active self-managers, possessing requisite skills, knowledge, and confidence to communicate about their pain experience, work with their health care providers, and self-manage their pain (as appropriate) throughout the course of their recovery. The SMArTVIEW intervention was designed accordingly over the course of 1 year; the key components are as follows.

## SMArTVIEW intervention

SMArTVIEW is a three-component intervention that combines postoperative remote automated monitoring (RAM) in hospital, hospital-to-home virtual recovery care and support, as well as self-management education.^17,57^ Postoperative RAM is supported by in-hospital wireless biosensors and a bedside monitor, which transmits and displays vital signs on a continual basis. Based on hospital early warning system parameters, notifications are sent to frontline nursing staff, calling for early attention to care should patients’ early warning scores escalate, indicating physiologic deterioration. Details of this early warning system and care escalation protocol are provided elsewhere.^57^ A recent state of the science paper on RAM by McGillion et al.^57^ identifies pain and related patient-reported outcomes, such as health-related quality of life, as a strategic direction for the development future of early warning systems, driven by machine learning algorithms.

Secondly, virtual hospital-to-home nurse-guided recovery support is facilitated through daily vital sign monitoring and daily video conferencing with specialized cardiac and vascular nurses, known as SMArTVIEW nurses (SVNs).When at home, patients complete three sets of vital signs per day for 30 days, which calculates a triage score in real time for the SVN nursing team. Higher triage scores indicate higher patient acuity. In addition, SVNs conduct standardized head-to-toe assessments and visualize incisional wounds.

Thirdly, patients engage in a 5-week tablet-based interactive self-management training program, titled “SMArTVIEW Restore and Recover” (R&R). SMArTVIEW R&R provides comprehensive education on postoperative recovery pathways (i.e., pain management, medication, nutrition, wound care, etc.) as well as facilitates forums to engage in peer support and reflective behavior. Patients complete approximately 1–2 h per week on self-directed self-management training.

### Clinical approach

The SMArTVIEW intervention is delivered by a speciality team of cardiac- or vascular-trained registered nurses to support the hospital-to-home intervention. Upon hire, SVNs are introduced to the intervention and provided a comprehensive review of evidence-based resources to teach/reinforce cardiac and major vascular surgical recovery pathways, patient assessments—including pain management—and common postoperative complications. When executing the SMArTVIEW hospital-to-home intervention, the SVNs complete a full head-to-toe remote assessment using video-based teleconferencing daily. In accordance with the Registered Nurses’ Association of Ontario’s best practice guideline *Assessment and Management of Pain*,^58^ SVNs gather and explore each patient’s previous pain history, sensory characteristics of pain, psychosocial impact, and effective interventions used in the past to manage pain to comprehensively understand each individual’s self-report. SVNs have access to patient data pertaining to their in-hospital stays though charting, including pain ratings and assessments, but SVNs also complete their own assessments to inform their care plans as a part of the SMArTVIEW protocol. Pain intensity is scored on a daily basis using an NRS (0–10; 0 = *no pain*, 10 = *worst imaginable pain*) and pain interference captured using the Brief Pain Inventory–Short Form on hospital discharge, day 7, and day 14 of the 30-day program. The SVN team dedicates a proportion of each daily video assessment strictly to address postoperative pain, intervening when pain escalates beyond a mild intensity score (NRS ≥ 4/10). Medication reconciliations are also completed on days 3, 10, 17, 24, and 30 wherein SVNs and patients conduct a comprehensive medication review. All prescribed medications, including analgesics, are reviewed with patients to ensure safe and appropriate medication administration, as well as optimal effectiveness to ensure that postoperative pain remains in the mild (NRS < 4/10) to no pain range. Using clinical judgment, SVNs suggest evidence-based over-the-counter medications that are safe for cardiac and major vascular surgical patients. Patients are given the opportunity to openly discuss concerns and experiences in order for the SVNs to make tailored pain management recommendations, including pharmacological and nonpharmacological, fluid management, wound management, education, and psychosocial support, to best meet the needs of the SMArTVIEW patients.

### SMArTVIEW self-management training

Ample literature,^59–63^ across a vast array of chronic diseases, has demonstrated a paradigm shift in health care that focuses attention away from clinician-based to patient-centered health care management and, more important, emphasizing patient self-management by way of self-management training. This shift of focus addresses a primary barrier to postoperative pain management by means of promoting patient education to facilitate patients’ competence in becoming actively engaged in their recovery.

In order to adequately prepare cardiac surgical patients for recovery at home, SVNs introduce SMArTVIEW R&R on day 2 to 3 of their hospital stay. As a core component of the SMArTVIEW intervention, SMArTVIEW R&R is a virtual tablet-based self-management program delivered over a 5-week period, beginning while in hospital (week 0) to 30 days postoperative (week 4). SMArTVIEW R&R was co-designed with patients to ensure that patient needs were met, with pain education being a primary concern following cardiac and major vascular surgery. Both content and process elements of R&R are grounded in the fear avoidance beliefs model, which shows how postoperative pain and recovery-related perceptions can lead to fear, hypervigilance, avoidance, disability, and depression.^64^ To mitigate recovery-related perceptions, the online interactive elements as well as self-efficacy-enhancing features of SMArTVIEW R&R have been adapted from the Help to Overcome Problems (HOPE) and iHOPE programs based out of Coventry University. HOPE and iHOPE^65–68^ are uniquely grounded in both positive psychology and cognitive behavioral therapy theory and incorporate evidence-based cognitive behavioral therapy and positive psychological activities such as goal setting and action planning, identifying personal strengths, scheduling activity and rest periods, mindfulness, relaxation training, and reviewing successes. Feasibility trials have shown that HOPE and iHOPE have the potential to improve important health-related quality of life outcomes for people living with and affected by a range of long-term conditions.^65–67^ In collaboration with Coventry University, content was strategically timed to correlate with the appropriate recovery stage of the patient, peer-based interaction by way of “gratitude diary entries,” and weekly goal setting.

**Figure 1.**
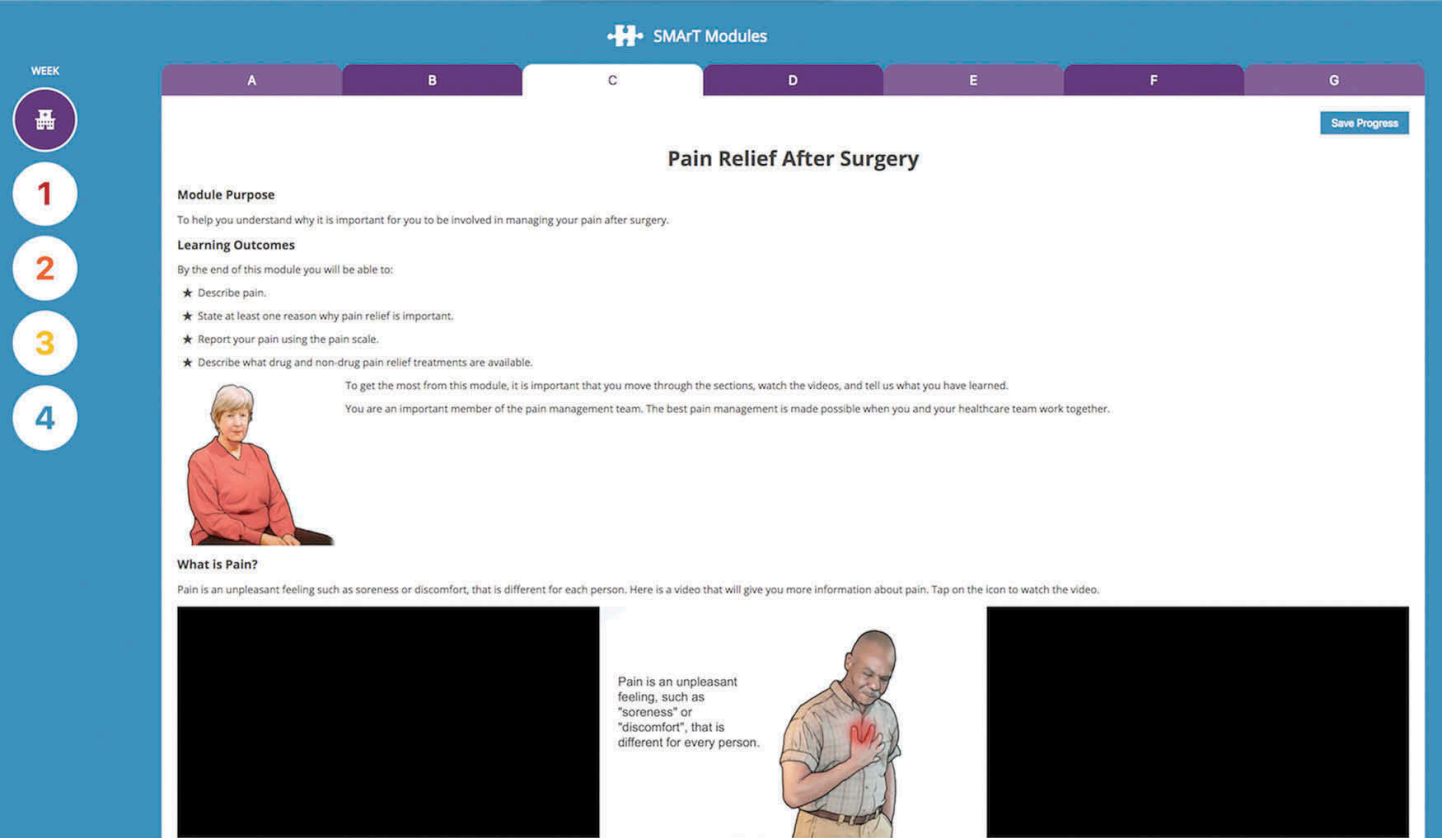
SMArTVIEW Restore & Recover.

Across these elements, acute postoperative pain is addressed through both clinical and self-management approaches. Pain management is introduced in week 0, prior to discharge, as Activity 3 “Pain Relief After Surgery” (Figure 1) and is re-established in week 2, Activity 2 “Common Concerns About Pain” (Table 1). Beyond the provision of educational materials, the curriculum is designed to provide patients with requisite cognitive, emotional, and behavioral skills to manage their postoperative pain and recovery experience in a productive and positive way, leading to optimal functioning. To this end, the program design also draws upon Bandura’s self-efficacy theory, with respect to four key elements of perceived self-efficacy, as follows:

**Table 1.** SMArTVIEW R&R curriculum.

	Hospital	Home			
	Week 0	Week 1	Week 2	Week 3	Week 4
Orientation to the SMArTVIEW self-management program	Activity 1	●	●	●	●
e-TrAC orientation	Activity 2	●	●	●	●
Pain relief after surgery	Activity 3	●	●	●	●
Managing your pain medication	Activity 4	●	●	●	●
Better breathing after surgery	Activity 5	●	●	●	●
Using your mind to help you heal	Activity 6	●	●	●	●
Gratitude	Activity 7	●	●	●	●
Managing your regular medications		Activity 1	●	●	●
Managing your postoperative wounds		Activity 2	●	●	●
Weight tracking for fluid management after cardiac surgery		Activity 3	●	●	●
Listening to your heart		Activity 4	●	●	●
Getting a good night’s sleep after CaVs surgery		Activity 5	●	●	●
Goal setting and action planning		Activity 6	●	●	●
Goal setting feedback			Activity 1	●	●
Common concerns about pain			Activity 2	●	●
Fear avoidance			Activity3	●	●
Mindfulness/relaxation			Activity 4	●	●
Physical activity and exercise			Activity 5	●	●
Preventing falls during recovery			Activity 6	●	●
Goal setting and action plan			Activity 7	●	●
Goal setting feedback				Activity 1	●
Nutrition for recovery				Activity 2	●
Instilling hope and other positive emotions after CaVs surgery				Activity 3	●
Dealing with low mood and stress during recovery				Activity 4	●
Saving your energy				Activity 5	●
Goal setting and action plan				Activity 6	●
Goal setting feedback					Activity 1
Staying active during recovery					Activity 2
Healthy eating for life					Activity 3
Communication skills—family, health care professional, and organization					Activity 4
Sexuality and intimacy					Activity 5
Dealing with setbacks during recovery and moving forward to lead a happy, flourishing life					Activity 6

R&R = Restore & Recover; e-TrAC = Electronic Transition to Ambulatory Care; CaVs = Cardiac and Vascular Surgery.

1. Mastery experience—Provision of opportunities to rehearse and apply self-management skills repeatedly in order to achieve success.
2. Vicarious learning—Watching and learning from the success of other patients in the program as well as by the example of the SMArTVIEW nurses.
3. Verbal persuasion—Provision of an accountability structure that facilitates regular participant debriefing regarding successes or challenges in meeting weekly self-management goals.
4. Physiological and emotional state—Allowing for daily check-in with the SMArTVIEW nurse about participant symptoms and mood, which can impact self-management goals.^69^

SVNs routinely assist patients through SMArTVIEW R&R in a coaching capacity and specifically monitor and follow up on R&R progress on days 3, 10, 17, 24, and 30 of the intervention.

In conclusion, patient, health care provider, and systems-level barriers have challenged the delivery and execution of postoperative pain assessment and management. Innovative approaches to address these barriers are a current challenge to health care providers and researchers alike. SMArTVIEW is spearheading this paradigm shift within clinical research to address barriers that impair effective postoperative pain management by actively engaging health care providers and patients in an accessible format (i.e., digital health solution) to give primacy to the need of postoperative pain assessment and management. It is our hope that the SMArTVIEW in-hospital monitoring and hospital-to-home nursing care support will not only help patients on an individual basis to better manage postoperative pain using best evidence grounded in research but also influence systems-level care by way of understanding how institutions can allocate resources more usefully to better meet the needs of patients following cardiac and major vascular surgery. Trial results will be disseminated upon trial completion.
